# Sex-Specific Limitations in Physical Health in Primary Adrenal Insufficiency

**DOI:** 10.3389/fendo.2021.718660

**Published:** 2021-10-18

**Authors:** Nora Møller Didriksen, Åse Bjorvatn Sævik, Linn Solveig Sortland, Marianne Øksnes, Eystein Sverre Husebye

**Affiliations:** ^1^ Department of Clinical Science, University of Bergen, Bergen, Norway; ^2^ K.G. Jebsen Center for Autoimmune Disorders, University of Bergen, Bergen, Norway; ^3^ National Centre for Emergency Primary Health Care, NORCE Norwegian Research Centre, Bergen, Norway; ^4^ Department of Medicine, Haukeland University Hospital, Bergen, Norway

**Keywords:** PAI, primary adrenal insufficiency, Addison disease, quality of life, QoL, RAND-36, physical health

## Abstract

**Background:**

Patients with primary adrenal insufficiency (PAI) suffer reduced quality of life (QoL), but comparisons with large-scale normative data are scarce. The clinical characteristics associated with reduced QoL are largely unknown.

**Methods:**

Cross-sectional data on clinical characteristics and QoL scores from 494 patients were included. QoL was measured using RAND-36 (generic) and AddiQoL (-30 and -8, disease-specific). RAND-36 is reported as subdomain scores as well as physical (PCS) and metal (MCS) summary scores and compared with normative data.

**Results:**

Perception of physical role was consistently decreased across age groups in patients with PAI compared with normative data [75 (0-100) *vs.* 100 (50-100), p<0.001]. Men with PAI reported significantly lower scores for social functioning [88 (75-100) *vs.* 100 (75-100), p<0.001], as well as for vitality and physical role. In women, the greatest impairment was seen in physical role [50 (0-100) *vs.* 100 (50-100), p<0.001], followed by social functioning, vitality, physical function, general health, mental health, and emotional role. Overall, better QoL was associated with male sex (AddiQoL-30: 89 ± 13 *vs.* 82 ± 13, p<0.002), younger age (e.g. 20-29 *vs.* 80-89 years: PCS 59 [50-62] *vs.* 46 [37-53], p<0.001), autoimmune etiology [PCS: 53 (45-59) *vs.*. 45 (38-54), p<0.001], and absence of autoimmune comorbidity [PCS: 54 (45-59) *vs.* 50 (43-58), p<0.001]. There were no significant differences in QoL scores between different doses or dosing regimens of glucocorticoid or mineralocorticoid replacement.

**Conclusion:**

QoL is reduced in patients with PAI, especially perception of physical role in women and social functioning in men. Among patients with PAI, female sex, higher age, non-autoimmune etiology, and autoimmune comorbidity was associated with lower QoL-scores.

## Introduction

Patients with primary adrenal insufficiency (PAI) depend on daily replacement of glucocorticoids and mineralocorticoids to survive. Yet current treatment strategies fail to restore full health evident by increased cardiometabolic risk, increased morbidity, and lower quality of life (1–4).

Although many studies have shown reduced quality of life (QoL) in PAI, valid data to explain these findings have been hindered by small sample sizes. Suggested influencing factors include sex, personality, autoimmune comorbidity, as well as dosing regimens, and modes of delivery of corticosteroids (5). To date, the majority of studies have focused on the latter, as treatment constitutes a modifiable variable with potential to improve QoL.

Published data point to a possible link between the daily glucocorticoid dosage and QoL scores. A chronic too high dosage could result in a slow decline in QoL scores over time, similar to findings in patients with autonomous cortisol production (6). Similarly, too low dosages could lead to fatigue and a higher frequency of adrenal crisis, and therefore worsened QoL (7, 8). Unfortunately, we have no perfect way of evaluating whether a patient receives the optimal dosage of glucocorticoids. Treatment surveillance is therefore mainly assessed using clinical signs of hypocortisolism (fatigue, abdominal or muscular pain, weight loss, hyperpigmentation) and hypercortisolism (weight gain, metabolic complications, psychiatric symptoms) (4).

Conventional glucocorticoid treatment with oral hydrocortisone or cortisone acetate is unable to imitate the circadian and ultradian variation in glucocorticoid secretion. As a result, patients may experience enlarged peaks and exaggerated troughs of glucocorticoid levels throughout the day (9, 10). Continuous subcutaneous hydrocortisone infusions (CSHI) by pump can imitate the physiological circadian profile of glucocorticoids, yet current data are insufficient to conclude on any effects on quality of life (QoL) (10–12). One would think that dividing the daily dosage into smaller doses during the day could improve health related QoL, although evidence to support this is scarce (13). Conversely, patients on extended-release hydrocortisone with only one cortisol peak tend to score better than conventional thrice daily hydrocortisone (14).

In PAI, the mineralocorticoid deficiency is replaced with fludrocortisone. Unfortunately, many are undertreated, evident by persistent light-headedness, salt cravings, postural hypotension and electrolyte disturbances (hyponatremia, hyperkalemia) (4, 15). In contrast, overtreatment may present as peripheral edema and hypertension (4, 15). Emerging evidence, including a clinical study of patients with PAI, suggested that the fludrocortisone dosage could affect QoL as the mineralocorticoid receptor plays a crucial role for cognition and mood (16).

To overcome previous shortcomings, we here use one of the world’s largest registries on PAI to investigate how clinical characteristics and associated diseases impact on QoL and compare the findings with normative data.

## Material and Methods

### Patients and Ethical Approval

The study participants were recruited from the Norwegian registry for organ-specific autoimmune diseases (ROAS). Cross-sectional data on QoL were available for 494 patients from 2002-2015. When multiple QoL scores were available from the same patient, the most recent was used. All patients enrolled in ROAS have provided written informed consent that the registered data may be used for research purposes. The study was approved by the regional committee for medical and health professional research-ethics: 2019/1146/REK-Nord.

### Normative Data

The normative data were calculated from a reference population previously drawn randomly from the National Population Registry, as part of a national postal survey, “Norwegian Level of Living Survey” in 2002-2003 (17). Among 9 675 invited subjects, 6 193 agreed to participate, yielding a response rate of 64%. Of these, 5290 completed the quality-of-life questionnaire and were included in the present analysis. Statistics Norway and The Norwegian Institute of Public Health were responsible for the data collection and Institute of Health and Society at University of Oslo, provided funding. Anonymized data were prepared and made available from The Norwegian Center for Research Data (NSD). All analyses and interpretations presented here are performed by the listed authors.

### Patient Characteristics

For each of the included patients, we noted the following clinical data: sex, age (by decade), disease duration, isolated PAI or presence of autoimmune polyendocrine syndrome type 1 (APS-1) or 2 (APS-2), presence of 21-hydroxylase autoantibodies (21OH-ab), type of glucocorticoid replacement, daily dosage of glucocorticoid replacement in mg hydrocortisone equivalents [using the standard ratio hydrocortisone:cortisone acetate 4:5 (18)], daily dosage of fludrocortisone replacement in mg, as well as any disability benefit. Any adrenal androgen replacement was initially noted, but not included in the statistical analyses due to few users (n=20).

### Quality of Life Questionnaires

QoL was assessed by two validated questionnaires, one generic (RAND-36) and one specific for PAI (AddiQoL-30 and -8). Common for both is that a higher score indicates better health-related quality of life. RAND-36, a license free version of SF-36, is a multi-purpose 36 question health survey with eight subdomains: physical functioning (PF), role-physical (RP), bodily pain (BP), general health (GH), vitality (V), social functioning (SF), role-emotional (RE) and mental health (MH) (19). Here, we present the results for each subdomain as well as summary scores of physical health (physical component summary; PCS) and mental health (mental component summary; MCS) (20, 21). The summary scores have been developed to simplify the analysis and interpretation of the results (22), but have not yet been investigated in patients with PAI. The subdomain scores were calculated in accordance with the RAND-36 scoring algorithm (19). For the summary scores, we chose the standard (US) orthogonal factor for correction to obtain uncorrelated PCS and MCS scores (20, 21). Of note, the algorithms described above for scoring subdomains and summary scores were used both for patient and normative data. AddiQoL has been validated and translated into several languages (23). AddiQoL-30 contains 30 questions made for assessment of four subdimensions (fatigue, emotions, symptoms, and miscellaneous which involves intercurrent disease, sleep, and sexuality) in patients with PAI (11, 23). AddiQoL-8 is a short-version of AddiQoL-30 that focuses on fatigue and energy level (23).

### Statistics

Normal distribution was evaluated using the Shapiro-Wilks test, and parametric and nonparametric tests were chosen as appropriate and reported as mean ± SD and median [interquartile range, IQR], respectively. The QoL scores are presented for the whole patient cohort and men and women separately, as well as subgroup analyses among patients with autoimmune etiology. To compare subgroups, we used the t-test or Mann-Whitney U test and Kruskal Wallis test, as appropriate. If one or more of the included variables lacked normal distribution, a nonparametric test was chosen. A p-value < 0.010 was defined as statistically significant.

## Results

Patient characteristics are presented in **Table 1**. In short, there was a predominance of female patients (61%) and autoimmune etiology (84%); the median age group was 50-59 years, and the median disease duration 11 [4-24] years.

**Table 1:** Patient characteristics.

No. patient visits (females)	494 (303)
No. RAND-36 measurements (PCS and MCS)	482
No. AddiQoL measurements (AddiQoL-8 and -30)	72
Age in decade years, median [IQR]	50-59 years [40-79]
Disease duration in years, median [IQR]	11 [4-24]
Positive for 21-hydroxylase autoantibodies, no. (%)	421 (85)
Presence of autoimmune comorbidity, no. (%)	293 (59)
APS-1, no. (%)	19 (4)
Hydrocortisone daily equivalent in mg, median [IQR]	30 [20-30]
Fludrocortisone daily dose in mg, median [IQR]	0.100 [0.100-0.100]

### RAND-36 Subdomains

**Figure 1:**
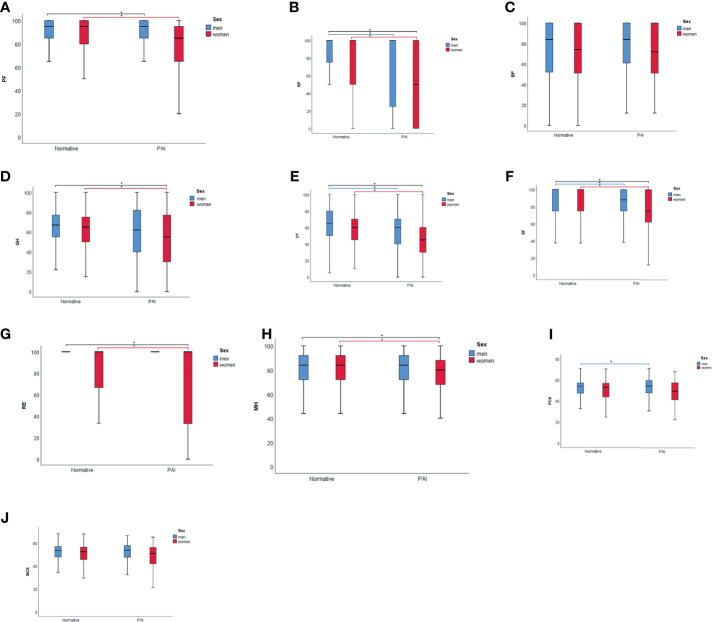
Scores for physical functions (PF) **(A)**, role physical (RP) **(B)**, bodily pain (BP) **(C)**, general health (GH) **(D)**, vitality (VT) **(E)**, social function (SF) **(F)**, role emotional (RE) **(G)**, mental health (MH) **(H)**, physical component summary (PCS) **(I)**, and mental component summary (MCS) **(J)** in men (blue) and women (red) for normative data and patients with primary adrenal insufficiency (PAI). *p < 0.010.

Patients with PAI had significantly lower scores for physical role (RP) in particular, but also for social function (SF), vitality (VT), general health (GH), physical function (PF), mental health (MH), and emotional role (RE) compared with normative data (in descending order of magnitude) (**Table 2**). The same was seen in men for SF, VT, and RP, and in women for RP, SF, VT, PF, GH, MH, and RE (**Figure 1**).

**Table 2:** RAND-36 subdomain scores in patients (Men, Women, and Total) with PAI compared with normative data.

	PF	RP	BP	GH	VT	SF	RE	MH
**Total**								
**PAI**	90	75	74	57	50	88	100	80
Median [IQR]	[72-95]	[0-100]	[51-100]	[35-77]	[30-65]	[62-100]	[67-100]	[68-88]
**Normative**	95	100	84	65	60	100	100	84
Median [IQR]	[85-100]	[50-100]	[51-100]	[50-75]	[45-75]	[75-100]	[100-100]	[72-92]
**p-value**	<0.001*	<0.001*	0.566	<0.001*	<0.001*	<0.001*	<0.001*	0.001*
**Men**								
**PAI**	95	100	84	62	60	88	100	84
Median [IQR]	[85-100]	[25-100]	[61-100]	[40-82]	[40-70]	[75-100]	[84-100]	[72-92]
**Normative**	95	100	84	67	65	100	100	84
Median [IQR]	[85-100]	[75-100]	[52-100]	[55-77]	[50-80]	[75-100]	[100-100]	[72-92]
p-value	0.060	<0.001*	0.193	0.071	<0.001*	0.001*	0.209	0.270
**Women**								
**PAI**	85	50	72	55	45	75	100	80
Median [IQR]	[65-95]	[0-100]	[51-100]	[30-77]	[30-60]	[62-100]	[33-100]	[67-88]
**Normative**	95	100	74	65	60	100	100	84
Median [IQR]	[80-100]	[50-100]	[51-100]	[50-75]	[45-70]	[75-100]	[67-100]	[72-92]
**p-value**	<0.001*	<0.001*	0.208	<0.001*	<0.001*	<0.001*	<0.001*	0.002*

PAI, primary adrenal insufficiency; IQR, interquartile range; PF, physical functioning; RP, role physical; BP, bodily pain; GH, general health; VT, vitality; SF, social functioning; RE, role emotional; MH, mental health. *; statistically significant defined as p < 0.010.

When comparing scores in corresponding age groups, RP was the most consistently decreased subdomain in PAI compared with normative data, significant for all age groups between 20 and 59 years. VT scores were significantly reduced for age groups 30-59 years, MH for 40-69 years, SF for 30-59 years, RE for 20-29 years, and 40-59 years, GH for 40-49 years, and PF for 40-49 years (**Supplementary Table 1**). For the oldest age groups, there were no significant differences in RAND-36 subdomain scores compared with normative data, except for better PF in patients aged 80-89.

### PCS and MCS Summary Scores

PCS and MCS scores were lower in patients with PAI as well as in the subgroup of females but did not significantly differ compared with normative data (**Table 3**). Statistical significance was, however, reached for PCS scores (but not MCS) in men, with slightly higher PCS score in PAI compared with normative data [54 (48-60) *vs.* 53 (48-57), p<0.005].

**Table 3:** Quality of life in men and women with PAI compared to normative data.

		Total	Men	Women	p-value
**Patients with PAI**					
	AddiQoL-30 mean ± SD (no)	83 ± 14 (72)	89 ± 13 (29)	80 ± 13 (43)	0.005*
	AddiQoL-8 mean ± SD (no)	20 ± 5 (72)	22 ± 4 (29)	18 ± 5 (43)	0.003*
	PCS median [IQR] (no)	52 [44-59] (482)	54 [48-60] (184)	49 [41-57] (298)	<0.001*
	MCS median [IQR] (no)	52 [44-57] (482)	54 [48-58] (184)	51 [42-56] (298)	0.002*
**Normative data**					
	PCS median [IQR] (no)	53 [47-57] (5048)	53 [48-57] (2466)	52 [46-56] (2582)	<0.001*
	MCS median [IQR] (no)	54 [46-57] (5048)	54 [47-57] (2466)	53 [44-57] (2582)	<0.001*

PAI, primary adrenal insufficiency; PCS, physical component summary; MCS, mental component summary. *; statistically significant defined as p < 0.010.

### Sex and Age

**Figure 2:**
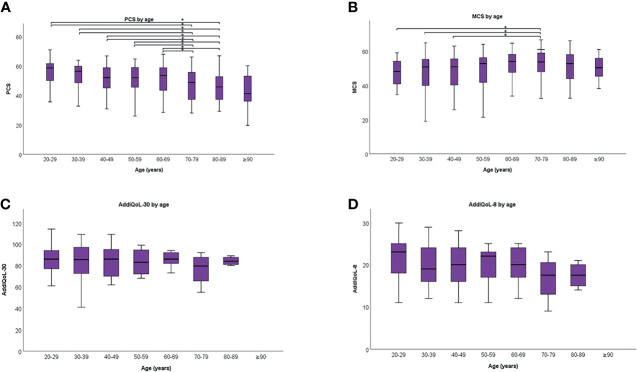
Physical component summary (PCS) **(A)**, mental component summary (MCS) **(B)**, AddiQol-30 **(C)**, and AddiQoL-8 **(D)** scores in different age groups. The box marks the interquartile range, black horizontal line the median, and the whiskers the range. *p < 0.010.

**Figure 3:**
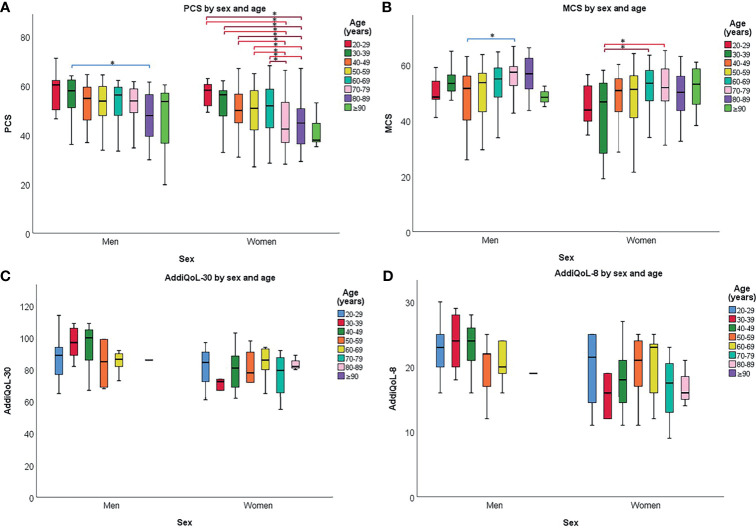
Physical component summary (PCS) **(A)**, mental component summary (MCS) **(B)**, AddiQoL-30 **(C)**, and AddiQoL-8 **(D)** scores in different age groups for men and women separately. The box marks the interquartile range, black horizontal line the median, and the whiskers the range. *p < 0.010.

Men reported significantly better QoL than women, evident by higher scores in AddiQoL-30, AddiQoL-8, MCS, and PCS (**Table 3**). Changes in PCS, MCS, and AddiQoL scores with age are shown in **Figures 2** and **3** and statistical comparison with normative data for PCS and MCS listed in **Supplementary Table 2**. In short, PCS scores were significantly higher in patients with PAI aged 20-39 compared with normative data, whereas MCS scores were significantly lower in patients with PAI for age groups 20-49. The opposite was seen for age groups 70-79, where patients with PAI reported significantly lower PCS scores but higher MCS scores compared with normative data. MCS scores were also significantly higher in patients with PAI ≥ 80 years old.

PCS scores significantly declined with age (**Figure 2A**). The trend remained significant in men and women when analyzed separately (**Figure 3A**). In contrast, MSC scores were significantly higher in patients aged 70-79 years compared with those 20-39 and 50-59 years old (**Figure 2B**). Similar trends were seen in men and women when analyzed separately (**Figure 3B**). There were no significant differences in AddiQoL-scores between age groups (**Figures 2C, D**, **3C, D**).

### Disease Duration

Patients diagnosed between 11-20 years ago, had higher PCS scores than patients with at least 20 years disease duration [11-20 years *vs.* >20 years: 54 (46-60) *vs.* 50 (38-57), p<0.001]. Opposite, MCS scores were lower in patients diagnosed between 2 and 5 years ago compared with patients with at least 20 years disease duration [2-5 years *vs.* >20 years: 51 (40-55) *vs.* 54 (47-58), p<0.002].

### Etiology and Comorbidity

Patients positive for 21OH-ab had significantly higher PCS scores compared those without 21OH-ab [53 (45-59) *vs.* 45 (38-54), p<0.001]. Scores for PF and RP were also higher [90 (75-100) *vs.* 75 (55-95), p<0.001 and 75 (0-100) *vs.* 25 (0-100), p<0.001, respectively]. However, patients negative for 21-hydroxylase autoantibodies were significantly older (median age 70-79 *vs.* 50-59 years, p<0.001).

For the subgroup of patients positive for 21OH-ab, scores for PCS, MCS, AddiQoL-30, and AddiQoL-8 remained significantly higher in men compared with women [PCS: 56 (48-60) *vs.* 50 (42-57), p<0.001, MCS: 54 (48-58) *vs.* 51 (43-56), p<0.009, AddiQoL-30: 91 (83-102) *vs.* 80 (72-91), p<0.002, AddiQoL-8: 22 (20-26) *vs.* 18 (16-23), p<0.005]. In addition, PCS scores were significantly higher in patients without any autoimmune comorbidity [56 (48-60) *vs.* 51 (44-58), p<0.001], and in younger compared with older age groups (e.g. 20-29 *vs.* 80-89 years: 59 (50-62) *vs.* 46 (38-56), p<0.001]. In contrast, MCS scores where significantly lower in age group 20-29 years compared with 70-79 years [48 (40-54) *vs.* 54 (48-59), p<0.006].

### Replacement Medication

Patients taking less than 15 mg hydrocortisone equivalents daily reported significantly higher MCS scores compared with those taking more than 35 mg [55 (53-57) *vs.* 49 (36-53), p<0.009]. Otherwise, there were no significant differences in RAND-36 subdomains, PCS, MCS, or AddiQoL scores and the daily dose or dosing regimen of glucocorticoid or mineralocorticoid replacement (data not shown).

### Disability Benefit

There were no significant differences in QoL scores between patients who received disability benefits compared with those who did not (data not shown).

## Discussion

In one of the largest studies to date on QoL in PAI, we show that RP is the most consistently impaired, especially in female patients. Lower QoL outcome is associated with female sex, high age, and autoimmune comorbidities, but no significant differences were found for modifiable parameters such as daily doses or dosing regimens of glucocorticoid or mineralocorticoid replacement.

Strikingly, female patients with PAI aged 30-49 had 50% lower RP scores than the background population, a score corresponding to normative data for 70-79-year-old individuals (RP=50), indicating that their disease severely influenced their working ability and daily activities (24, 25). This was not, however, seen in male patients where only patients aged 20-29 and 40-49 years reported significantly lower RP scores. In fact, male patients aged 70-79 years had higher RP scores compared with normative data, whereas female patients still reported half the RP score compared to controls. Although these latter observations did not reach statistical significance, they add to the impression that QoL in women with PAI is more affected than in men. Indeed, the differences in median scores for RAND-36 subdomains were in general greater in women than in men compared with normative data. At the same time, it must be noted that a substantial number of patients reported QoL equal to the normative data, evident by the wide interquartile range in scores, highlighting the heterogeneity in QoL outcome in both men and women with PAI.

Somewhat surprising, we found slightly higher PCS scores in men with PAI compared with normative data, and otherwise no significant differences in PCS or MCS scores. This may be related to the heterogeneity in patient characteristics, as statistical significance was reached between PAI and normative data for some of the age groups. Worth mentioning, the normative data used in the present study were collected at the start of the collection period for patient data, yet a study comparing Norwegian QoL reference values from 1996, 2002, and 2015 concluded that QoL scores remained stable across this time period despite societal changes (26). We are therefore confident that the normative data are representative for the entire study period.

For both sexes, PCS declined with age, in line with previous studies on PAI and the general population (23, 26–28). For MCS, however, patients in some of the older age groups had higher scores compared with younger age groups. We acknowledge that this finding may be an unfavorable consequence of using an orthogonal factor for calculation of summary scores as done in the present study. Here, three of the physical-related subdomains (PF, RP, and BP) are negatively weighed in the calculation of MCS. In other words, a high degree of physical disability reported (which is to be expected with older age) may artificially increase MCS, as described in other studies as well (29). This may also in part explain our findings of higher PCS scores in patients diagnosed 11-20 years vs. more than 20 years ago, whereas MCS scores were lower in patients diagnosed 2-5 years vs. more than 20 years ago. Alternatively, the observed increase in MCS could also be explained by response shift – that is, changes in the patients’ internal standards, values, and thoughts on QoL after receiving a diagnosis or experiencing an adrenal crisis (30).

Although we found a beneficial effect of autoimmune etiology on physical QoL in PAI, this may be confounded by significantly higher age in patients without autoimmune cause. Indeed, the differences in QoL between patients with and without autoimmune etiology disappeared when stratifying for age, although this might be partly due to loss of statistical power (data not shown). In the current study, most patients without 21OH-ab were categorized as “idiopathic” with regards to etiology or were bilaterally adrenalectomized. For the 44 patients with idiopathic cause, autoimmune origin may still be possible, suggested by the conspicuously high number of patients with idiopathic cause and autoimmune comorbidities (n=18). In the 19 bilaterally adrenalectomized patients, we find it plausible that QoL could be lower since many of them were treated for Cushing’s syndrome, a disease known to significantly impact negatively on QoL (31).

To our surprise, we could barely find any significant impact of glucocorticoid (GC) or mineralocorticoid (MC) replacement on QoL, neither for daily doses nor dosing regimens, as previously reported (13, 32). The only significant finding was higher MCS in patients taking low doses of hydrocortisone equivalents (<15 mg daily) compared with supraphysiological doses (> 35 mg daily). This indicates that the magnitude of GC exposure is not the main driver of QoL outcome in PAI. Instead, the inability of standard replacement treatment to replicate the circadian and ultradian rhythmicity of cortisol exposure in healthy individuals may be more important, as suggested by some studies on continuous subcutaneous hydrocortisone infusions and extended-release hydrocortisone (4, 31). Unfortunately, we were unable to explore the significance of such near-physiological GC exposure on QoL outcome in the present study but encourage future investigations in this direction. As a final note, striving for a physiological GC replacement dose is still of utmost importance to avoid the high symptom-burden of hypocortisolism and to reduce the risk of metabolic and cardiovascular morbidity due to hypercortisolism (4).

We suspect our study was not sufficiently powered to detect possible differences in QoL related to MC doses as this information was only available for 118 patients, of whom 67 used 0.1 mg. Previously, van der Valk and colleagues demonstrated impaired QoL in patients with PAI not taking any MC replacement, illustrating the importance of sufficient replacement (33). Furthermore, evidence from both animal and human studies, demonstrate that occupation of mineralocorticoid receptors in the brain is essential for normal cognitive function and mood (16).

Our findings paint a different picture of QoL in patients with PAI than the nation-wide investigation performed by Løvås et al. two decades ago, where GH and VT were the most consistently impaired subdomains (32). When comparing median scores, the present patient cohort report higher median scores for GH, but lower for VT (and BP). The difference is most distinct in female patients, where we found lower scores for SF as well, but also higher scores for PF, RP, and RE. In men, we found higher scores for GH, VT, and MH, and no subdomains with lower scores than reported by Løvås et al. We find this particularly interesting as our patient cohort were on average 10 years older and had a greater proportion of females (61% vs. 55%), factors that are associated with worse QoL. These observations give reason for careful optimism, as there seems to have been an improvement over the last two decades. Whether the different findings represent true changes or not, and if so, what the causes are, remains to be investigated. Nevertheless, as median scores for normative data have remained stable over the past decades (34), we are inclined to think there has been a true improvement in patient QoL, possibly related to better patient care.

Apart from an expected difference between men and women, we did not find any associations between clinical characteristics and disease-specific QoL as measured by AddiQoL-30 and -8. We suspect this may relate to the small number of AddiQoL scores included since the AddiQoL questionnaire was introduced late in the study period. This is a limitation of the current study. Likewise, small sample sizes complicate the interpretation of subgroup analyses for some of the age groups, as there were few patients and controls among the youngest and the oldest. Furthermore, as few of the included patients used other GC treatment modalities than conventional oral cortisone acetate or hydrocortisone, we were unable to explore any impact on QoL by e.g. extended-release hydrocortisone or subcutaneous hydrocortisone infusion by pump. Finally, the cross-sectional study design prevented us from drawing conclusions on causality. Indeed, a longitudinal study should be performed to address whether different interventions may improve QoL in PAI.

In conclusion, we show that men and women with PAI report different limitations in QoL compared with normative data, with perception of physical role being the most impaired in women and social functioning in men. Overall, better QoL outcome is associated with male sex, lower age, autoimmune etiology, and absence of autoimmune comorbidity, but no differences were detected for modifiable parameters such as glucocorticoid or mineralocorticoid replacement doses.

## Data Availability Statement

The raw data supporting the conclusions of this article will be made available by the authors, without undue reservation.

## Ethics Statement

The studies involving human participants were reviewed and approved by the regional committee for medical and health professional research-ethics: 2019/1146/REK-Nord. The patients/participants provided their written informed consent to participate in this study.

## Author Contributions

EH proposed the study, and together with ND, ÅS, LS, and MØ, drafted the study plan. LS collected the data, and ND performed the statistical analyses. ND and ÅS wrote the initial draft. ND, ÅS, MØ, and ES all contributed to the interpretation of the study and to further drafts. All authors contributed to the article and approved the submitted version.

## Funding

The study was supported by the Regional Health Authorities of Western Norway, The University of Bergen, The Research Council of Norway (no.288022), The Novonordisk Foundation, and Stiftelsen Kristian Gerhard Jebsen.

## Conflict of Interest

The authors declare that the research was conducted in the absence of any commercial or financial relationships that could be construed as a potential conflict of interest.

## Publisher’s Note

All claims expressed in this article are solely those of the authors and do not necessarily represent those of their affiliated organizations, or those of the publisher, the editors and the reviewers. Any product that may be evaluated in this article, or claim that may be made by its manufacturer, is not guaranteed or endorsed by the publisher.
