# Supplementary material for: Sex-Specific Limitations in Physical Health in Primary Adrenal Insufficiency
Source: Front Endocrinol (Lausanne). 2021 Oct 18;12:718660. doi: 10.3389/fendo.2021.718660 (PMC8558514; doi:10.3389/fendo.2021.718660)
Supplement: Supplementary file 1 [file Table_1.docx]

#### Supplementary appendix

##### Table 1

RAND-36 subdomain scores for age subgroups in men and women as well as for the total patient group with PAI compared with normative data. *p<0.010.

| **Age Group** |  |  | **n** | **PF** | **RP** | **BP** | **GH** | **VT** | **SF** | **RE** | **MH** |
| --- | --- | --- | --- | --- | --- | --- | --- | --- | --- | --- | --- |
| **<20** | **Total** |  |  |  |  |  |  |  |  |  |  |
|  | **PAI**  Median [IQR] |  | 4 | 95  [54-97] | 100  [25-100] | 92  [21-100] | 56  [46-85] | 65  [56-70] | 82  [38-97] | 67  [33-100] | 74  [62-89] |
|  | **Normative**  Median [IQR] |  | 293 | 100  [95-100] | 100  [100-100] | 84  [72-100] | 70  [50-77] | 55  [43-70] | 100  [75-100] | 100  [100-100] | 80  [68-84] |
|  | **p-value** |  |  | 0.210 | 0.719 | 0.822 | 0.529 | 0.322 | 0.307 | 0.145 | 0.735 |
|  | **Men** |  |  |  |  |  |  |  |  |  |  |
|  | **PAI**  Median [IQR] |  | 3 | 95  [40-95] | 100  [0-100] | 84  [0-84] | 47  [45-47] | 60  [55-60] | 88  [25-88] | 33  [33-33] | 68  [60-68] |
|  | **Normative**  Median [IQE] |  | 150 | 100  [95-100] | 100  [100-100] | 84  [74-100] | 70  [60-80] | 60  [45-75] | 100  [75-100] | 100  [100-100] | 80  [72-88] |
|  | **p-value** |  |  | 0.292 | 0.420 | 0.635 | 0.080 | 0.822 | 0.441 | 0.017 | 0.141 |
|  | **Women** |  |  |  |  |  |  |  |  |  |  |
|  | **PAI**  Median [IQR] |  | 1 | 95 | 100 | 100 | 92 | 70 | 75 | 100 | 92 |
|  | **Normative**  Median [IQR] |  | 143 | 100  [95-100] | 100  [94-100] | 84  [72-100] | 70  [55-75] | 55  [40-65] | 88  [75-100] | 100  [67-100] | 76  [68-84] |
|  | **p-value** |  |  | 0.643 | 0.755 | 0.333 | 0.139 | 0.374 | 0.500 | 0.694 | 0.139 |
| **20-29** | **Total** |  |  |  |  |  |  |  |  |  |  |
|  | **PAI**  Median [IQR] |  | 21 | 95  [90-100] | 75  [50-100] | 100  [68-100] | 62  [42-86] | 60  [33-65] | 75  [63-100] | 68  [33-100] | 80  [66-88] |
|  | **Normative**  Median [IQR] |  | 717 | 100  [95-100] | 100  [100-100] | 84  [64-100] | 70  [60-80] | 60  [45-70] | 100  [75-100] | 100  [100-100] | 80  [72-88] |
|  | **p-value** |  |  | 0.058 | 0.001* | 0.286 | 0.199 | 0.046 | 0.020 | 0.001* | 0.326 |
|  | **Men** |  |  |  |  |  |  |  |  |  |  |
|  | **PAI**  Median [IQR] |  | 9 | 100  [93-100] | 100  [63-100] | 100  [62-100] | 82  [50-96] | 60  [35-70] | 100  [75-100] | 100  [68-100] | 80  [68-86] |
|  | **Normative**  Median [IQR] |  | 321 | 100  [95-100] | 100  [100-100] | 84  [62-100] | 70  [60-80] | 65  [50-75] | 100  [88-100] | 100  [100-100] | 80  [72-88] |
|  | **p-value** |  |  | 0.490 | 0.116 | 0.351 | 0.348 | 0.179 | 0.967 | 0.394 | 0.745 |
|  | **Women** |  |  |  |  |  |  |  |  |  |  |
|  | **PAI**  Median [IQR] |  | 12 | 95  [79-100] | 75  [25-100] | 92  [77-100] | 60  [42-66] | 58  [24-64] | 75  [38-97] | 67  [33-100] | 76  [65-88] |
|  | **Normative**  Median [IQR] |  | 396 | 100  [95-100] | 100  [100-100] | 84  [72-100] | 70  [57-80] | 60  [45-70] | 100  [75-100] | 100  [100-100] | 80  [72-88] |
|  | **p-value** |  |  | 0.057 | 0.002* | 0.527 | 0.013 | 0.154 | 0.002* | <0.001* | 0.313 |
| **30-39** | **Total** |  |  |  |  |  |  |  |  |  |  |
|  | **PAI**  Median [IQR] |  | 45 | 95  [83-100] | 75  [13-100] | 100  [62-100] | 62  [35-82] | 50  [30-68] | 75  [50-100] | 100  [50-100] | 80  [62-86] |
|  | **Normative**  Median [IQR] |  | 1001 | 95  [95-100] | 100  [100-100] | 84  [62-100] | 70  [55-80] | 65  [50-75] | 100  [75-100] | 100  [100-100] | 84  [72-92] |
|  | **p-value** |  |  | 0.021 | <0.001* | 0.390 | 0.055 | 0.001* | <0.001* | 0.025 | 0.023 |
|  | **Men** |  |  |  |  |  |  |  |  |  |  |
|  | **PAI**  Median [IQR] |  | 22 | 98  [90-100] | 100  [69-100] | 100  [70-100] | 70  [55-89] | 58  [45-75] | 94  [69-100] | 100  [100-100] | 84  [72-88] |
|  | **Normative**  Median [IQR] |  | 476 | 100  [95-100] | 100  [100-100] | 84  [62-100] | 70  [55-80] | 65  [50-75] | 100  [88-100] | 100  [100-100] | 84  [76-92] |
|  | **p-value** |  |  | 0.485 | 0.073 | 0.194 | 0.687 | 0.296 | 0.163 | 0.436 | 0.880 |
|  | **Women** |  |  |  |  |  |  |  |  |  |  |
|  | **PAI**  Median [IQR] |  | 23 | 90  [70-100] | 50  [0-100] | 72  [51-100] | 50  [25-72] | 40  [25-60] | 75  [50-75] | 100  [0-100] | 76  [48-84] |
|  | **Normative**  Median [IQR] |  | 525 | 95  [93-100] | 100  [75-100] | 84  [62-100] | 70  [55-80] | 60  [45-70] | 100  [75-100] | 100  [100-100] | 84  [72-88] |
|  | **p-value** |  |  | 0.010* | <0.001* | 0.931 | 0.003* | <0.001* | <0.001* | <0.001* | 0.002* |
| **40-49** | **Total** |  |  |  |  |  |  |  |  |  |  |
|  | **PAI**  Median [IQR] |  | 92 | 95  [80-100] | 75  [0-100] | 72  [51-100] | 57  [30-77] | 50  [26-60] | 75  [62-100] | 100  [67-100] | 80  [68-88] |
|  | **Normative**  Median [IQR] |  | 1069 | 95  [90-100] | 100  [75-100] | 84  [51-100] | 70  [55-80] | 65  [50-75] | 100  [75-100] | 100  [100-100] | 84  [72-92] |
|  | **p-value** |  |  | 0.004* | <0.001* | 0.353 | <0.001* | <0.001* | <0.001* | <0.001* | 0.002* |
|  | **Men** |  |  |  |  |  |  |  |  |  |  |
|  | **PAI**  Median [IQR] |  | 37 | 95  [90-100] | 75  [25-100] | 72  [51-100] | 52  [31-71] | 50  [30-70] | 88  [62-100] | 100  [67-100] | 80  [66-88] |
|  | **Normative**  Median [IQR] |  | 541 | 95  [90-100] | 100  [75-100] | 84  [61-100] | 70  [55-80] | 65  [55-80] | 100  [75-100] | 100  [100-100] | 84  [76-92] |
|  | **p-value** |  |  | 0.719 | 0.001* | 0.311 | <0.001* | <0.001* | 0.020 | 0.010* | 0.026 |
|  | **Women** |  |  |  |  |  |  |  |  |  |  |
|  | **PAI**  Median [IQR] |  | 55 | 90  [75-95] | 50  [0-100] | 72  [51-100] | 62  [30-82] | 40  [25-60] | 75  [63-100] | 100  [67-100] | 80  [72-88] |
|  | **Normative**  Median [IQR] |  | 528 | 95  [90-100] | 100  [75-100] | 74  [51-100] | 70  [55-77] | 65  [45-75] | 100  [75-100] | 100  [100-100] | 84  [72-92] |
|  | **p-value** |  |  | 0.001* | <0.001* | 0.865 | 0.039 | <0.001* | <0.001* | 0.001* | 0.041 |
| **50-59** | **Total** |  |  |  |  |  |  |  |  |  |  |
|  | **PAI**  Median [IQR] |  | 104 | 90  [75-100] | 75  [0-100] | 74  [51-100] | 59  [32-82] | 45  [30-65] | 88  [62-100] | 100  [42-100] | 80  [68-88] |
|  | **Normative**  Median [IQR] |  | 967 | 95  [80-95] | 100  [50-100] | 72  [51-100] | 65  [50-75] | 65  [50-80] | 100  [75-100] | 100  [100-100] | 84  [72-92] |
|  | **p-value** |  |  | 0.459 | <0.001* | 0.450 | 0.316 | <0.001* | 0.001* | 0.008* | 0.005* |
|  | **Men** |  |  |  |  |  |  |  |  |  |  |
|  | **PAI**  Median [IQR] |  | 39 | 95  [85-100] | 100  [10-100] | 84  [52-100] | 62  [32-82] | 55  [35-75] | 88  [75-100] | 100  [67-100] | 80  [64-88] |
|  | **Normative**  Median [IQR] |  | 481 | 95  [85-100] | 100  [75-100] | 80  [51-100] | 65  [50-75] | 70  [55-80] | 100  [75-100] | 100  [100-100] | 88  [76-92] |
|  | **p-value** |  |  | 0.310 | 0.035 | 0.588 | 0.618 | 0.009* | 0.276 | 0.263 | 0.072 |
|  | **Women** |  |  |  |  |  |  |  |  |  |  |
|  | **PAI**  Median [IQR] |  | 65 | 85  [70-95] | 75  [0-100] | 74  [42-100] | 57  [29-82] | 45  [30-60] | 75  [50-100] | 100  [33-100] | 80  [68-88] |
|  | **Normative**  Median [IQR] |  | 486 | 90  [75-95] | 100  [50-100] | 62  [41-100] | 65  [45-75] | 60  [45-75] | 100  [75-100] | 100  [100-100] | 84  [72-92] |
|  | **p-value** |  |  | 0.203 | <0.001* | 0.340 | 0.462 | <0.001* | 0.002* | 0.019 | 0.060 |
| **60-69** | **Total** |  |  |  |  |  |  |  |  |  |  |
|  | **PAI**  Median [IQR] |  | 93 | 90  [70-95] | 75  [25-100] | 74  [51-100] | 62  [39-81] | 55  [35-70] | 88  [75-100] | 100  [100-100] | 80  [68-92] |
|  | **Normative**  Median [IQR] |  | 661 | 85  [65-95] | 100  [25-100] | 64  [41-100] | 60  [45-75] | 65  [50-80] | 100  [75-100] | 100  [67-100] | 84  [72-92] |
|  | **p-value** |  |  | 0.015 | 0.246 | 0.037 | 0.821 | <0.001* | 0.012 | 0.261 | 0.092 |
|  | **Men** |  |  |  |  |  |  |  |  |  |  |
|  | **PAI**  Median [IQR] |  | 33 | 95  [80-100] | 100  [63-100] | 84  [62-100] | 67  [47-85] | 60  [40-70] | 88  [75-100] | 100  [100-100] | 84  [70-92] |
|  | **Normative**  Median [IQR] |  | 335 | 90  [75-95] | 100  [25-100] | 72  [51-100] | 65  [45-75] | 70  [50-80] | 100  [75-100] | 100  [100-100] | 88  [76-96] |
|  | **p-value** |  |  | 0.199 | 0.837 | 0.074 | 0.512 | 0.008* | 0.131 | 0.232 | 0.485 |
|  | **Women** |  |  |  |  |  |  |  |  |  |  |
|  | **PAI**  Median [IQR] |  | 60 | 90  [75-95] | 50  [0-100] | 74  [51-100] | 57  [32-79] | 50  [31-79] | 88  [66-100] | 100  [67-100] | 80  [68-88] |
|  | **Normative**  Median [IQR] |  | 326 | 85  [60-95] | 75  [25-100] | 62  [41-84] | 60  [40-75] | 60  [45-75] | 100  [75-100] | 100  [67-100] | 84  [68-92] |
|  | **p-value** |  |  | 0.006* | 0.212 | 0.093 | 0.942 | 0.012 | 0.074 | 0.323 | 0.297 |
| **70-79** | **Total** |  |  |  |  |  |  |  |  |  |  |
|  | **PAI**  Median [IQR] |  | 66 | 75  [55-95] | 50  [0-100] | 72  [41-100] | 57  [30-72] | 55  [40-70] | 88  [62-100] | 100  [33-100] | 84  [72-92] |
|  | **Normative**  Median [IQR] |  | 416 | 75  [50-90] | 50  [0-100] | 62  [41-100] | 55  [40-72] | 60  [41-75] | 88  [63-100] | 100  [33-100] | 84  [68-92] |
|  | **p-value** |  |  | 0.199 | 0.957 | 0.564 | 0.534 | 0.160 | 0.860 | 0.526 | 0.572 |
|  | **Men** |  |  |  |  |  |  |  |  |  |  |
|  | **PAI**  Median [IQR] |  | 23 | 90  [75-95] | 100  [50-100] | 84  [62-100] | 62  [52-77] | 65  [60-75] | 100  [62-100] | 100  [100-100] | 88  [84-92] |
|  | **Normative**  Median [IQR] |  | 195 | 80  [65-95] | 75  [0-100] | 72  [51-100] | 60  [40-73] | 65  [45-80] | 100  [75-100] | 100  [67-100] | 84  [76-96] |
|  | **p-value** |  |  | 0.033 | 0.124 | 0.067 | 0.214 | 0.607 | 0.947 | 0.061 | 0.594 |
|  | **Women** |  |  |  |  |  |  |  |  |  |  |
|  | **PAI**  Median [IQR] |  | 43 | 65  [50-85] | 25  [0-100] | 62  [41-84] | 45  [25-62] | 50  [35-60] | 88  [62-100] | 100  [0-100] | 84  [72-92] |
|  | **Normative**  Median [IQR] |  | 221 | 65  [41-85] | 50  [0-100] | 62  [41-100] | 55  [40-70] | 55  [39-70] | 88  [63-100] | 100  [0-100] | 80  [64-92] |
|  | **p-value** |  |  | 0.342 | 0.400 | 0.900 | 0.123 | 0.060 | 0.777 | 0.963 | 0.473 |
| **80-89** | **Total** |  |  |  |  |  |  |  |  |  |  |
|  | **PAI**  Median [IQR] |  | 49 | 70  [53-86] | 25  [0-100] | 62  [41-100] | 50  [35-74] | 50  [35-63] | 88  [62-100] | 100  [33-100] | 80  [64-90] |
|  | **Normative**  Median [IQR] |  | 151 | 55  [30-75] | 0  [0-75] | 52  [41-80] | 50  [35-65] | 50  [36-65] | 75  [50-100] | 67  [0-100] | 76  [60-92] |
|  | **p-value** |  |  | 0.003* | 0.031 | 0.056 | 0.516 | 0.419 | 0.070 | 0.033 | 0.443 |
|  | **Men** |  |  |  |  |  |  |  |  |  |  |
|  | **PAI**  Median [IQR] |  | 16 | 80  [61-90] | 25  [0-100] | 84  [54-100] | 65  [36-77] | 63  [35-78] | 94  [62-100] | 100  [75-100] | 86  [78-99] |
|  | **Normative**  Median [IQR] |  | 61 | 65  [48-80] | 0  [0-75] | 62  [41-84] | 50  [35-63] | 55  [40-75] | 88  [50-100] | 33  [0-100] | 80  [65-92] |
|  | **p-value** |  |  | 0.062 | 0.405 | 0.079 | 0.060 | 0.702 | 0.316 | 0.020 | 0.108 |
|  | **Women** |  |  |  |  |  |  |  |  |  |  |
|  | **PAI**  Median [IQR] |  | 33 | 60  [43-85] | 25  [0-100] | 62  [41-100] | 45  [32-61] | 45  [33-55] | 88  [56-100] | 67  [33-100] | 72  [62-86] |
|  | **Normative**  Median [IQR] |  | 90 | 45  [25-71] | 0  [0-63] | 51  [32-80] | 50  [38-65] | 50  [35-63] | 75  [50-100] | 67  [0-100] | 72  [54-92] |
|  | **p-value** |  |  | 0.005* | 0.036 | 0.171 | 0.550 | 0.309 | 0.126 | 0.289 | 0.878 |
| **>90** | **Total** |  |  |  |  |  |  |  |  |  |  |
|  | **PAI**  Median [IQR] |  | 8 | 58  [26-85] | 13  [0-65] | 68  [31-100] | 57  [29-73] | 53  [36-60] | 94  [60-100] | 50  [00-100] | 76  [60-88] |
|  | **Normative**  Median [IQR] |  | 13 | 43  [6-5] | 0  [0-38] | 51  [41-92] | 50  [25-62] | 55  [43-65] | 75  [31-100] | 100  [67-100] | 84  [74-96] |
|  | **p-value** |  |  | 0.207 | 0.696 | 0.804 | 0.697 | 0.50 | 0.210 | 0.161 | 0.140 |
|  | **Men** |  |  |  |  |  |  |  |  |  |  |
|  | **PAI**  Median [IQR] |  | 3 | 70  [20-95] | 25  [0-75] | 100  [2-100] | 62  [10-77] | 60  [10-60] | 88  [50-100] | 33  [0-67] | 76  [76-92] |
|  | **Normative**  Median [IQR] |  | 7 | 45  [4-63] | 0  [0-75] | 52  [40-84] | 60  [45-62] | 50  [40-65] | 75  [38-100] | 100  [33-100] | 84  [72-96] |
|  | **p-value** |  |  | 0.381 | 0.714 | 0.667 | 0.833 | 1.0 | 0.833 | 0.183 | 0.833 |
|  | **Women** |  |  |  |  |  |  |  |  |  |  |
|  | **PAI**  Median [IQR] |  | 5 | 50  [28-78] | 0  [0-63] | 52  [31-92] | 57  [3-67] | 45  [38-75] | 100  [69-100] | 100  [0-100] | 60  [58-84] |
|  | **Normative**  Median [IQR] |  | 6 | 43  [9-63] | 13  [0-25] | 46  [36-100] | 35  [19-57] | 58  [50-68] | 75  [22-91] | 100  [92-100] | 86  [62-96] |
|  | **p-value** |  |  | 0.537 | 1.0 | 1.0 | 0.329 | 0.662 | 0.126 | 0.429 | 0.247 |

PF; physical functioning, RP; role physical, BP; bodily pain, GH; general health, VT; vitality, SF; social functioning, RE; role emotional, MH; mental health. *; statistically significant defined as p<0.010.

##### Table 2

RAND-36 summary scores (PCS: A, MCS: B) for age subgroups in men and women as well as for the total patient group with PAI compared with normative data.

1. PCS

| **Age Group** |  | **n** | **PCS** |
| --- | --- | --- | --- |
| **<20** | **Total** |  |  |
|  | **PAI**  Median [IQR] | 4 | 59 [38-64] |
|  | **Normative**  Median [IQR] | 287 | 50 [44-54] |
|  | **p-value** |  | 0.096 |
|  | **Men** |  |  |
|  | **PAI**  Median [IQR] | 3 | 57 [32-57] |
|  | **Normative**  Median [IQR] | 146 | 51 [47-54] |
|  | **p-value** |  | 0.395 |
|  | **Women** |  |  |
|  | **PAI**  Median [IQR] | 1 | 60 |
|  | **Normative**  Median [IQR] | 141 | 48 [41-52] |
|  | **p-value** |  | 0.056 |
| **20-29** | **Total** |  |  |
|  | **PAI**  Median [IQR] | 21 | 59 [50-62] |
|  | **Normative**  Median [IQR] | 690 | 52 [46-55] |
|  | **p-value** |  | <0.001* |
|  | **Men** |  |  |
|  | **PAI**  Median [IQR] | 9 | 60 [50-63] |
|  | **Normative**  Median [IQR] | 307 | 52 [46-55] |
|  | **p-value** |  | 0.007* |
|  | **Women** |  |  |
|  | **PAI**  Median [IQR] | 12 | 58 [50-61] |
|  | **Normative**  Median [IQR] | 383 | 52 [46-55] |
|  | **p-value** |  | 0.005* |
| **30-39** | **Total** |  |  |
|  | **PAI**  Median [IQR] | 44 | 57 [49-60] |
|  | **Normative**  Median [IQR] | 981 | 52 [47-56] |
|  | **p-value** |  | 0.001* |
|  | **Men** |  |  |
|  | **PAI**  Median [IQR] | 21 | 58 [49-62] |
|  | **Normative**  Median [IQR] | 471 | 53 [47-56] |
|  | **p-value** |  | 0.002* |
|  | **Women** |  |  |
|  | **PAI**  Median [IQR] | 23 | 56 [47-58] |
|  | **Normative**  Median [IQR] | 510 | 51 [46-56] |
|  | **p-value** |  | 0.069 |
| **40-49** | **Total** |  |  |
|  | **PAI**  Median [IQR] | 92 | 52 [45-59] |
|  | **Normative**  Median [IQR] | 1034 | 53 [48-57] |
|  | **p-value** |  | 0.915 |
|  | **Men** |  |  |
|  | **PAI**  Median [IQR] | 37 | 55 [46-59] |
|  | **Normative**  Median [IQR] | 525 | 53 [49-59] |
|  | **p-value** |  | 0.367 |
|  | **Women** |  |  |
|  | **PAI**  Median [IQR] | 55 | 50 [45-57] |
|  | **Normative**  Median [IQR] | 509 | 53 [48-56] |
|  | **p-value** |  | 0.455 |
| **50-59** | **Total** |  |  |
|  | **PAI**  Median [IQR] | 104 | 52 [46-59] |
|  | **Normative**  Median [IQR] | 932 | 54 [48-58] |
|  | **p-value** |  | 0.916 |
|  | **Men** |  |  |
|  | **PAI**  Median [IQR] | 39 | 54 [48-60] |
|  | **Normative**  Median [IQR] | 464 | 55 [49-58] |
|  | **p-value** |  | 0.444 |
|  | **Women** |  |  |
|  | **PAI**  Median [IQR] | 65 | 51 [42-58] |
|  | **Normative**  Median [IQR] | 468 | 53 [47-57] |
|  | **p-value** |  | 0.618 |
| **60-69** | **Total** |  |  |
|  | **PAI**  Median [IQR] | 93 | 54 [44-59] |
|  | **Normative**  Median [IQR] | 625 | 55 [49-58] |
|  | **p-value** |  | 0.291 |
|  | **Men** |  |  |
|  | **PAI**  Median [IQR] | 33 | 56 [47-60] |
|  | **Normative**  Median [IQR] | 317 | 55 [51-59] |
|  | **p-value** |  | 0.849 |
|  | **Women** |  |  |
|  | **PAI**  Median [IQR] | 60 | 52 [43-59] |
|  | **Normative**  Median [IQR] | 308 | 54 [45-58] |
|  | **p-value** |  | 0.447 |
| **70-79** | **Total** |  |  |
|  | **PAI**  Median [IQR] | 66 | 49 [37-56] |
|  | **Normative**  Median [IQR] | 367 | 54 [45-58] |
|  | **p-value** |  | 0.003* |
|  | **Men** |  |  |
|  | **PAI**  Median [IQR] | 23 | 54 [47-59] |
|  | **Normative**  Median [IQR] | 176 | 55 [48-59] |
|  | **p-value** |  | 0.717 |
|  | **Women** |  |  |
|  | **PAI**  Median [IQR] | 43 | 42 [37-53] |
|  | **Normative**  Median [IQR] | 191 | 52 [41-58] |
|  | **p-value** |  | 0.003* |
| **80-89** | **Total** |  |  |
|  | **PAI**  Median [IQR] | 49 | 46 [37-53] |
|  | **Normative**  Median [IQR] | 123 | 50 [42-57] |
|  | **p-value** |  | 0.021 |
|  | **Men** |  |  |
|  | **PAI**  Median [IQR] | 16 | 48 [39-56] |
|  | **Normative**  Median [IQR] | 55 | 47 [42-57] |
|  | **p-value** |  | 0.752 |
|  | **Women** |  |  |
|  | **PAI**  Median [IQR] | 33 | 45 [36-51] |
|  | **Normative**  Median [IQR] | 68 | 52 [43-58] |
|  | **p-value** |  | 0.010* |
| **>90** | **Total** |  |  |
|  | **PAI**  Median [IQR] | 8 | 41 [36-53] |
|  | **Normative**  Median [IQR] | 9 | 53 [44-62] |
|  | **p-value** |  | 0.167 |
|  | **Men** |  |  |
|  | **PAI**  Median [IQR] | 3 | 53 [20-62] |
|  | **Normative**  Median [IQR] | 5 | 52 [40-63] |
|  | **p-value** |  | 0.786 |
|  | **Women** |  |  |
|  | **PAI**  Median [IQR] | 5 | 38 [36-49] |
|  | **Normative**  Median [IQR] | 4 | 56 [43-61] |
|  | **p-value** |  | 0.111 |

1. MCS

| **Age Group** |  | **n** |  |
| --- | --- | --- | --- |
| **<20** | **Total** |  |  |
|  | **PAI**  Median [IQR] | 4 | 49 [40-55] |
|  | **Normative**  Median [IQR] | 287 | 56 [52-58] |
|  | **p-value** |  | 0.058 |
|  | **Men** |  |  |
|  | **PAI**  Median [IQR] | 3 | 43 [39-43] |
|  | **Normative**  Median [IQR] | 146 | 56 [52-58] |
|  | **p-value** |  | 0.044 |
|  | **Women** |  |  |
|  | **PAI**  Median [IQR] | 1 | 55 |
|  | **Normative**  Median [IQR] | 141 | 56 [52-58] |
|  | **p-value** |  | 0.789 |
| **20-29** | **Total** |  |  |
|  | **PAI**  Median [IQR] | 21 | 48 [40-54] |
|  | **Normative**  Median [IQR] | 690 | 56 [52-58] |
|  | **p-value** |  | <0.001* |
|  | **Men** |  |  |
|  | **PAI**  Median [IQR] | 9 | 48 [44-55] |
|  | **Normative**  Median [IQR] | 307 | 56 [52-58] |
|  | **p-value** |  | 0.015 |
|  | **Women** |  |  |
|  | **PAI**  Median [IQR] | 12 | 44 [40-56] |
|  | **Normative**  Median [IQR] | 383 | 55 [51-58] |
|  | **p-value** |  | <0.001* |
| **30-39** | **Total** |  |  |
|  | **PAI**  Median [IQR] | 44 | 51 [38-55] |
|  | **Normative**  Median [IQR] | 981 | 55 [50-58] |
|  | **p-value** |  | <0.001* |
|  | **Men** |  |  |
|  | **PAI**  Median [IQR] | 21 | 53 [49-56] |
|  | **Normative**  Median [IQR] | 471 | 55 [51-58 |
|  | **p-value** |  | 0.185 |
|  | **Women** |  |  |
|  | **PAI**  Median [IQR] | 23 | 47 [28-54] |
|  | **Normative**  Median [IQR] | 510 | 58 [56-58] |
|  | **p-value** |  | <0.001* |
| **40-49** | **Total** |  |  |
|  | **PAI**  Median [IQR] | 92 | 51 [40-56] |
|  | **Normative**  Median [IQR] | 1034 | 54 [48-57] |
|  | **p-value** |  | <0.001* |
|  | **Men** |  |  |
|  | **PAI**  Median [IQR] | 37 | 51 [40-56] |
|  | **Normative**  Median [IQR] | 525 | 55 [49-57] |
|  | **p-value** |  | 0.019 |
|  | **Women** |  |  |
|  | **PAI**  Median [IQR] | 55 | 51 [42-55] |
|  | **Normative**  Median [IQR] | 509 | 54 [47-57] |
|  | **p-value** |  | 0.009* |
| **50-59** | **Total** |  |  |
|  | **PAI**  Median [IQR] | 104 | 53 [41-56] |
|  | **Normative**  Median [IQR] | 932 | 52 [43-56] |
|  | **p-value** |  | 0.520 |
|  | **Men** |  |  |
|  | **PAI**  Median [IQR] | 39 | 54 [43-58] |
|  | **Normative**  Median [IQR] | 464 | 53 [46-56] |
|  | **p-value** |  | 0.446 |
|  | **Women** |  |  |
|  | **PAI**  Median [IQR] | 65 | 51 [41-56] |
|  | **Normative**  Median [IQR] | 468 | 51 [41-56] |
|  | **p-value** |  | 0.571 |
| **60-69** | **Total** |  |  |
|  | **PAI**  Median [IQR] | 93 | 54 [48-58] |
|  | **Normative**  Median [IQR] | 625 | 49 [39-55] |
|  | **p-value** |  | <0.001* |
|  | **Men** |  |  |
|  | **PAI**  Median [IQR] | 33 | 55 [48-59] |
|  | **Normative**  Median [IQR] | 317 | 51 [41-55] |
|  | **p-value** |  | 0.001* |
|  | **Women** |  |  |
|  | **PAI**  Median [IQR] | 60 | 53 [47-58] |
|  | **Normative**  Median [IQR] | 308 | 48 [37-54] |
|  | **p-value** |  | <0.001* |
| **70-79** | **Total** |  |  |
|  | **PAI**  Median [IQR] | 66 | 54 [48-59] |
|  | **Normative**  Median [IQR] | 367 | 47 [36-52] |
|  | **p-value** |  | <0.001* |
|  | **Men** |  |  |
|  | **PAI**  Median [IQR] | 23 | 57 [52-60] |
|  | **Normative**  Median [IQR] | 176 | 47 [40-53] |
|  | **p-value** |  | <0.001* |
|  | **Women** |  |  |
|  | **PAI**  Median [IQR] | 43 | 52 [47-59] |
|  | **Normative**  Median [IQR] | 191 | 42 [35-51] |
|  | **p-value** |  | <0.001* |
| **80-89** | **Total** |  |  |
|  | **PAI**  Median [IQR] | 53 | 53 [44-58] |
|  | **Normative**  Median [IQR] | 123 | 38 [33-46] |
|  | **p-value** |  | <0.001* |
|  | **Men** |  |  |
|  | **PAI**  Median [IQR] | 16 | 57 [51-63] |
|  | **Normative**  Median [IQR] | 55 | 41 [34-47] |
|  | **p-value** |  | <0.001* |
|  | **Women** |  |  |
|  | **PAI**  Median [IQR] | 33 | 50 [43-56] |
|  | **Normative**  Median [IQR] | 68 | 37 [29-44] |
|  | **p-value** |  | <0.001* |
| **>90** | **Total** |  |  |
|  | **PAI**  Median [IQR] | 8 | 50 [45-57] |
|  | **Normative**  Median [IQR] | 9 | 33 [23-36] |
|  | **p-value** |  | 0.001* |
|  | **Men** |  |  |
|  | **PAI**  Median [IQR] | 3 | 48 [45-48] |
|  | **Normative**  Median [IQR] | 5 | 33 [22-41] |
|  | **p-value** |  | 0.071 |
|  | **Women** |  |  |
|  | **PAI**  Median [IQR] | 5 | 53 [42-60] |
|  | **Normative**  Median [IQR] | 4 | 34 [26-36] |
|  | **p-value** |  | 0.016 |
